# The Na^+^/K^+^‐ATPase β1 Subunit is a Kidney ADP‐Ribosyl Cyclase

**DOI:** 10.1096/fj.202502065RR

**Published:** 2025-10-14

**Authors:** Tae‐Sik Nam, Seong‐Kyu Choe, Uh‐Hyun Kim

**Affiliations:** ^1^ Department of Biochemistry Wonkwang University Iksan Korea; ^2^ Department of Microbiology Wonkwang University Iksan Korea; ^3^ Sarcopenia Total Solution Center, School of Medicine Wonkwang University Iksan Korea

**Keywords:** β1 subunit of Na, K‐ATPase, ADP‐ribosyl cyclase, angiotensin II, Ca^2+^ signaling, kidney

## Abstract

ADP‐ribosyl cyclase (ARC) produces a Ca^2+^‐mobilizing second messenger, cyclic ADP‐ribose (cADPR), from NAD. In this study, we purified an ARC from rat kidney, which was identified as the β1 subunit of Na, K‐ATPase (Atp1b1). Recombinant Atp1b1 exhibited ARC activity and generated cADPR. Knockdown of Atp1b1 in mouse mesangial cells (MES‐13) inhibited angiotensin II (AngII)‐induced cADPR synthesis and the subsequent long‐lasting Ca^2+^ signals. These findings demonstrate that Atp1b1 is coupled with the AngII receptor and is activated to produce cADPR, thereby mediating Ca^2+^ signaling in the kidney.

## Graphical abstract

This study highlights that the β1 subunit of Na, K‐ATPase, known as Atp1b1, functions as an ADP‐ribosyl cyclase in rat kidney (kARC). We found that reducing Atp1b1 expression in mouse mesangial cells suppressed angiotensin II (AngII)–induced synthesis of cyclic ADP‐ribose (cADPR) and the associated sustained Ca^2+^ signaling. This suggests that Atp1b1 interacts with the AngII receptor (AT1R) and becomes activated to produce cADPR, which then mediates Ca^2+^ signaling pathways crucial for kidney function.

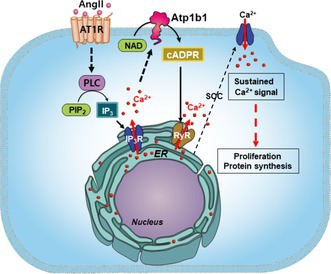

## 1 Introduction

Ca^2+^ mobilization is a universal signaling mechanism responsible for regulating virtually all aspects of cellular activities [1]. Three major Ca^2+^‐mobilizing second messengers–inositol 1,4,5‐trisphosphate, cyclic ADP‐ribose (cADPR), and nicotinic acid adenine dinucleotide phosphate (NAADP)–have been identified [2, 3]. The first ADP‐ribosyl cyclase (ARC) enzyme, which catalyzes the conversion of NAD to cADPR, was purified and cloned from the mollusk *Aplysia californica* [4, 5]. It exhibited sequence similarity to the human lymphocyte antigen CD38, which later emerged as the best characterized mammalian ARC [6]. Both CD38 and the *Aplysia* ARC are multifunctional enzymes that not only produce cADPR by cyclizing NAD but also generate NAADP via the base‐exchange reaction [7, 8].

Accumulating evidence indicates that additional ARCs exist beyond CD38 in mammalian tissues [9]. To this end, we purified ARC from rat kidney and provided evidence for the presence of a distinct ARC in the kidney. Furthermore, our results demonstrated that the kidney ARC (kARC) is the β1 subunit of Na, K‐ATPase (Atp1b1) [10].

## 2 Materials and Methods

### 2.1 Animals

C57BL/6 mice and Sprague‐Dawley rats were purchased from Orient (Seongnam, KOREA). Mice were inbred and housed under specific pathogen‐free conditions at Wonkwang University Medical School. All procedures complied with NIH guidelines and were approved by the Institutional Animal Care and Use Committee (WKU22‐04).

### 2.2 Cell Culture

HEK293 cells were cultured in Dulbecco's Modified Eagle's Medium (DMEM) supplemented with 10% fetal bovine serum (FBS), 100 units/mL penicillin, and 100 μg/mL streptomycin. Mouse mesangial cell line (MES‐13) was obtained from American Type Culture Collection (ATCC; Rockville, MD) and maintained in DMEM containing 5% FBS, 100 units/mL penicillin, and 100 μg/mL streptomycin in a humidified incubator at 37°C in the presence of 5% CO_2_ and 95% air. Cells were passaged three times per week.

### 2.3 Purification of ARC From Rat Kidney

Rat kidneys (250–300 g) were washed with ice‐cold PBS, minced in a lysis buffer (20 mM Tris–HCl, pH 7.2, 150 mM NaCl, and 1 mM PMSF), and homogenized in an ice bath. After centrifugation at 500 × *g* for 10 min, the supernatant was ultracentrifuged at 100 000 × *g* for 1 h. Pellets were solubilized with 1% NP‐40 in lysis buffer for 1 h. CD157 was removed via ultracentrifugation at 100 000 × *g* for 1 h; the supernatant was used for ARC purification. CD38 passed through DEAE‐Sepharose, while the ARC bound. Fractions eluted with 1 M NaCl from the DEAE were loaded onto Cibacron Blue 3GA agarose, onto Ni^2+^‐chelating Sepharose, and eluted with 200 mM imidazole. The purified ARC was separated on SDS–PAGE (11%) according to the method described by Xie et al. [11], and the activity band was eluted, concentrated using acetone precipitation, and stained with Coomassie brilliant blue.

### 2.4 Mass Spectrometry

Q‐TOP analysis of trypsin‐digested peptide fragments of purified ARC was performed by PROTEIN WORKS, Daejeon, Korea.

### 2.5 Plasmids and Transfection

The vector pCMV6‐Entry was used, and the construct consisted of the full‐length coding sequences of mouse Atp1b1, Sarm1, or CD38 cloned into it. HEK293 cells were transfected with the plasmids using Lipofectamine 2000 transfection reagent (Invitrogen) according to the manufacturer's instructions. Cells were used 24 h post‐transfection.

### 2.6 Measurement of Intracellular cADPR Concentration ([cADPR]_i_) and NAADP Concentration ([NAADP]_i_)

[cADPR]_i_ and [NAADP]_i_ were measured by the cyclic enzymatic assay as described previously [12, 13].

### 2.7 Measurement of ARC Activity

ARC activity was determined by measuring cyclic GDP‐ribose (cGDPR) using NGD^+^ as a substrate, as described previously [14]. Fluorescence of cGDPR was determined at 297‐nm excitation/410‐nm emission (F2500, Hitachi, Tokyo, Japan).

### 2.8 RT‐PCR (Quantitative PCR)

Complementary DNA (cDNA) was prepared from the RNA samples isolated from mouse tissues and was then used as the template in PCR reactions using specific primers for Atp1b1 (forward primer 5′‐atggggaagggggttggacgagacaa‐3′; reverse primer 5′‐gtagtaggtttccttctccacccagccg‐3′, yielding 915 bp PCR product). The amplified products were separated on 1.5% agarose gels.

### 2.9 Western Blot Analysis

Lysed cells, tissues and purified samples were reduced and separated by SDS‐PAGE (12% gel). The resolved proteins were transferred to a nitrocellulose membrane (Bio‐Rad). Antibodies used were: Atp1b1, Proteintech; Atp1a1, Santa cruz; actin Santa cruz; CD157, Santa cruz; CD38, Santa cruz.

### 2.10 Measurement of [Ca^2+^]_i_

Changes in [Ca^2+^]_i_ in MES‐13 cells were assessed as previously described [15]. Cells were loaded with 5 μM Fluo‐4 AM in HBSS at 37°C for 40 min followed by three washes. Fluorescence changes were monitored at 488 nm excitation and 530 nm emission using a confocal microscope every 4 s for 400 s. [Ca^2+^]_i_ was calculated using Tsien's equation [16].

### 2.11 Statistical Analysis

All data were analyzed using Student's *t* test or ANOVA, as appropriate. A *p* value of less than 0.05 was considered statistically significant.

## 3 Results and Discussion

**FIGURE 1:**
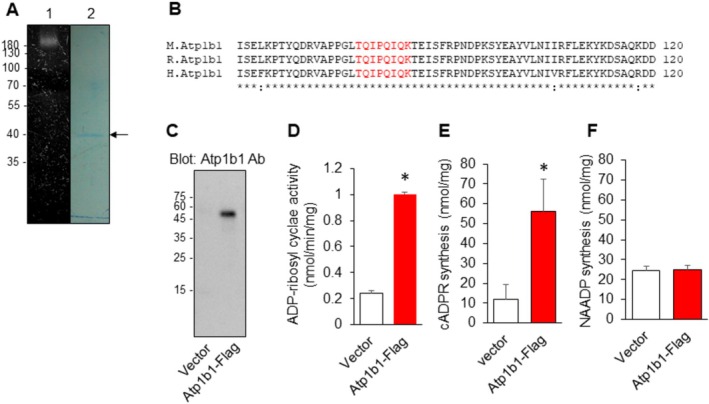
Identification of Atp1b1 as a novel kidney ARC (kARC). (A) Purification of the novel ARC from rat kidney. Visualization of the ARC activity using an in‐gel assay (lane 1, SDS‐PAGE under non‐reducing conditions) and Coomassie blue staining (lane 2, SDS‐PAGE under reducing conditions). Arrow indicates kARC. (B) Sequence alignment of the amino acids around ‘TQIPQIQK’ (in red) in mouse (M), rat (R), and human (H) Atp1b1, showing a perfect match among the three species. (C) Western blot analysis of the purified proteins using an anti‐Atp1b1 antibody. Atp1b1‐Flag was purified from HEK293 cells transfected with empty vector or Atp1b1‐Flag using anti‐Flag agarose. (D) ARC activity of the purified Atp1b1‐Flag protein. (E) cADPR synthesis of the purified Atp1b1‐Flag protein. (F) NAADP synthesis activity of Atp1b1‐Flag. *p < 0.05 versus vector.

Purification of kARC involved several chromatographic steps, including a DEAE‐Sepharose to remove CD38. In‐gel analysis of the final purified protein on an SDS‐PAGE under non‐reducing conditions showed an activity band at ~180 kDa (Figure 1A; lane 1). The eluted band under reducing conditions revealed a ~40 kDa protein (p40) (Figure 1A; lane 2), which was identified by Q‐TOF MS/MS as the peptide ‘TQIPQIQK’. This sequence corresponds to residues 78–85 in the β1 subunit of Na, K‐ATPase (Atp1b1) and is highly conserved across species (Figure 1B). Notably, Atp1b1 showed limited homology to known ARCs; only 14% and 15% amino acid sequence identity, and 28% and 29% similarity, with mouse CD38 and *Aplysia* ARC, respectively. To assess whether ATP1B1 possesses ARC activity, recombinant mouse ATP1B1‐Flag was expressed and shown to have ARC activity as well as the ability to synthesize cADPR, but not NAADP (Figure 1C–F and Figure S1). When we compared the ARC activity of recombinant Atp1b1 with those of CD38 and Sarm1 [17], we found that kARC activity was similar to that of Sarm1, whereas CD38 activity was approximately 260‐fold higher than kARC activity (Figure S2). We further analyzed the activity of kARC against NAD and nicotinamide guanine dinucleotide (NGD) to catalyze its NADase and ARC reactions using the Michaelis–Menten equation. The Km values of kARC for NAD and NGD were 65.0 ± 10.69 and 24.97 ± 1.68 μM, respectively, while the maximum enzyme velocity (Vmax) was 3.18 ± 0.31 nmol/mg/min and 1.18 ± 0.015 nmol/mg/min, respectively. As previously suggested [18], the compound 4,4′‐Dihydroxyazobenzene (4DHAB) is a specific inhibitor of kidney ARC. We confirmed that 4DHAB inhibited recombinant kARC activity (Figure S3).

**FIGURE 2:**
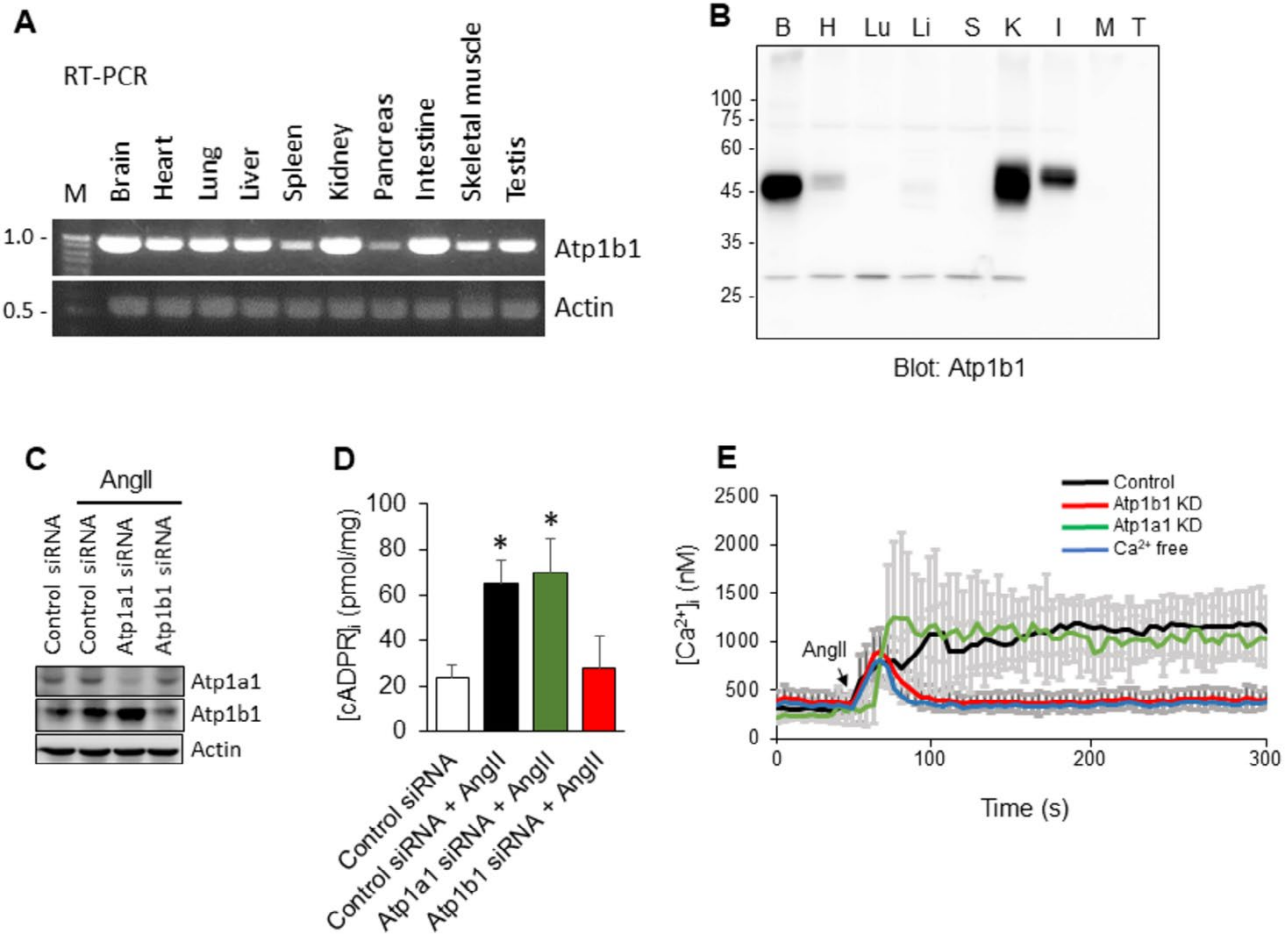
Expression of Atp1b1 in mouse tissues and the role of Atp1b1 in AngII‐mediated Ca^2+^ signaling in MES‐13 cells. (A) RT‐PCR analysis of Atp1b1 mRNA levels in various mouse tissues. Actin served as the housekeeping gene. (B) Western blot analysis of Atp1b1 protein levels across mouse tissues. Abbreviations: B, brain; H, heart; Lu, lung; Li, liver; S, spleen; K, kidney; I, intestine; M, skeletal muscle; T, testis. (C) Western blot of Atp1a1 and Atp1b1 in MES‐13 cells transfected with control siRNA, Atp1a1 siRNA, or Atp1b1 siRNA. (D) AngII‐induced intracellular cADPR synthesis in MES‐13 cells transfected with control, Atp1a1, or Atp1b1 siRNA. (E) AngII‐induced Ca^2+^ signaling (*n* = 3) in MES‐13 cells transfected with control, Atp1a1, or Atp1b1 siRNA, or in the absence of extracellular Ca^2+^. *p < 0.05 versus control siRNA.

We next analyzed Atp1b1 mRNA expression across various rat tissues using RT‐PCR and detected a ~1 kb transcript in the brain, heart, lung, liver, spleen, kidney, pancreas, intestine, skeletal muscle, and testis (Figure 2A). Correspondingly, Atp1b1 protein expression was evaluated by Western blot in the same tissues, revealing a ~45 kDa protein predominantly in the kidney, brain and intestine (Figure 2B).

Previously, we demonstrated that AngII treatment of MES‐13 cells activates ARC to produce cADPR, leading to a sustained increase in [Ca^2+^]_i_ [15]. Based on this, we hypothesized that knockdown of Atp1b1 would abolish AngII‐induced cADPR synthesis and Ca^2+^ signaling in MES‐13 cells. Indeed, knockdown of Atp1b1 with specific siRNA completely blocked AngII‐induced cADPR production and the sustained [Ca^2+^]_i_ rise in MES‐13 cells (Figure 2A–C). In contrast, knockdown of Atp1a1, the α subunit of Na, K‐ATPase, did not affect AngII‐induced cADPR synthesis or Ca^2+^ signaling (Figure 2A–C). The absence of extracellular Ca^2+^ resulted in the complete suppression of the late phase of sustained [Ca^2+^]_i_ rise, which is attributable to store‐operated Ca^2+^ entry triggered by cADPR‐mediated depletion of endoplasmic reticulum Ca^2+^ stores [19] (Figure 2E). These findings suggest that Atp1b1, independently of Atp1a1, is functionally coupled to the AngII receptor and mediates cADPR production, which in turn evokes the sustained [Ca^2+^]_i_ rise observed in AngII‐induced Ca^2+^ signals in MES‐13 cells.

Similar to CD38, Atp1b1 is expressed on the cell surface as a type II transmembrane protein, with its catalytic C‐terminal domain facing extracellularly. This raises a topological problem: how can the ectoenzyme utilize intracellular substrate NAD to produce cADPR, which targets cytosolic channels? It is of interest to explore how Atp1b1 addresses this topological challenge [20].

Based on the base‐exchange activity for NAADP synthesis using cell lysates transfected with ATP1b1‐flag, ATP1b1 appears to have no NAADP‐synthesizing activity. To date, CD157 has been found to possess only cADPR‐synthesizing activity, but not NAADP‐synthesizing activity [21].

Together, the results show that kARC is Atp1b1, a new family of ARC that appears to be phylogenetically distant from the mammalian prototype CD38. Since kARC plays a central role in AngII‐induced pathogenesis in the kidney, such as in diabetic nephropathy [22], Atp1b1 may serve as a therapeutic target for the treatment of chronic kidney disease.

## Author Contributions

Uh‐Hyun Kim designed the research. Tae‐Sik Nam and Seong‐Kyu Choe performed experiments and analysis. Uh‐Hyun Kim reviewed and wrote the manuscript.

## Conflicts of Interest

The authors declare no conflicts of interest.

## Supporting information


**Figure S1:** Time course of cGDPR production by Atp1b1‐Flag protein. ARC activity was determined by measuring cGDPR using NGD+ as a substrate.
**Figure S2:** ADP‐ribosyl cyclase activities of the purified Atp1b1‐Flag, Sarm1‐Flag and CD38‐Flag proteins.
**Figure S3:** Effect of 4DHAB (200 μM) on ADP‐ribosyl cyclase activity of the purified Atp1b1‐Flag.

## Data Availability

The datasets used and/or analyzed during the current study are available from the corresponding author on reasonable request.

## References

[1] H. C. Lee , “Cyclic ADP‐Ribose and Nicotinic Acid Adenine Dinucleotide Phosphate (NAADP) as Messengers for Calcium Mobilization,” Journal of Biological Chemistry 287 (2012): 31633–31640. 22822066 10.1074/jbc.R112.349464 PMC3442497

[2] M. J. Berridge , “Unlocking the Secrets of Cell Signaling,” Annual Review of Physiology 67 (2005): 1–21. 10.1146/annurev.physiol.67.040103.152647 15709950

[3] U. H. Kim , “Roles of cADPR and NAADP in Pancreatic Beta Cell Signaling,” Cell Calcium 103 (2022): 102562. 35219154 10.1016/j.ceca.2022.102562

[4] H. C. Lee and R. Aarhus , “ADP‐Ribosyl Cyclase: An Enzyme That Cyclizes NAD^+^ Into a Calcium‐Mobilizing Metabolite,” Cell Regulation 2 (1991): 203–209. 1830494 10.1091/mbc.2.3.203 PMC361752

[5] D. L. Glick , M. R. Hellmich , S. Beushausen , et al., “Primary Structure of a Molluscan Egg‐Specific NADase, a Second‐Messenger Enzyme,” Cell Regulation 2 (1991): 211–218. 1650255 10.1091/mbc.2.3.211 PMC361754

[6] D. J. States , T. F. Walseth , and H. C. Lee , “Similarities in Amino Acid Sequences of Aplysia ADP‐Ribosyl Cyclase and Human Lymphocyte Antigen CD38,” Trends in Biochemical Sciences 17 (1992): 495. 10.1016/0968-0004(92)90337-9 1471258

[7] R. Aarhus , R. M. Graeff , D. M. Dickey , T. F. Walseth , and H. C. Lee , “ADP‐Ribosyl Cyclase and CD38 Catalyze the Synthesis of a Calcium Mobilizing Metabolite From NADP,” Journal of Biological Chemistry 270 (1995): 30327–30333. 8530456 10.1074/jbc.270.51.30327

[8] T. S. Nam , D. R. Park , S. Y. Rah , et al., “Interleukin‐8 Drives CD38 to Form NAADP From NADP and NAAD in the Endolysosomes to Mobilize Ca^2+^ and Effect Cell Migration,” FASEB Journal 34 (2020): 12565–12576. 32717131 10.1096/fj.202001249R

[9] S. Partida‐Sanchez , D. A. Cockayne , S. Monard , et al., “Cyclic ADP‐Ribose Production by CD38 Regulates Intracellular Calcium Release, Extracellular Calcium Influx and Chemotaxis in Neutrophils and Is Required for Bacterial Clearance In Vivo,” Nature Medicine 7 (2001): 1209–1216. 10.1038/nm1101-1209 11689885

[10] G. Blanco and R. W. Mercer , “Isozymes of the Na‐K‐ATPase: Heterogeneity in Structure, Diversity in Function,” American Journal of Physiology 275 (1998): F633–F650. 9815123 10.1152/ajprenal.1998.275.5.F633

[11] G. H. Xie , S. Y. Rah , S. J. Kim , et al., “ADP‐Ribosyl Cyclase Couples to Cyclic AMP Signaling in the Cardiomyocytes,” Biochemical and Biophysical Research Communications 330 (2005): 1290–1298. 15823583 10.1016/j.bbrc.2005.03.114

[12] R. Graeff and H. C. Lee , “A Novel Cycling Assay for Cellular cADP‐Ribose With Nanomolar Sensitivity,” Biochemical Journal 361 (2002): 379–384. 11772410 10.1042/bj3610379 PMC1222318

[13] R. Graeff and H. C. Lee , “A Novel Cycling Assay for Nicotinic Acid‐Adenine Dinucleotide Phosphate With Nanomolar Sensitivity,” Biochemical Journal 367 (2002): 163–168. 12117413 10.1042/BJ20020644 PMC1222877

[14] R. M. Graeff , T. F. Walseth , K. Fryxell , W. D. Branton , and H. C. Lee , “Enzymatic Synthesis and Characterizations of Cyclic GDP‐ Ribose. A Procedure for Distinguishing Enzymes With ADP‐ Ribosyl Cyclase Activity,” Journal of Biological Chemistry 269 (1994): 30260–30267. 7982936

[15] S. Y. Kim , R. Gul , S. Y. Rah , et al., “Molecular Mechanism of ADP‐Ribosyl Cyclase Activation in Angiotensin II Signaling in Murine Mesangial Cells,” American Journal of Physiology 294 (2008): F982–F989. 18272599 10.1152/ajprenal.00483.2007

[16] R. Y. Tsien , T. Pozzan , and T. J. Rink , “T‐Cell Mitogens Cause Early Changes in Cytoplasmic Free Ca^2+^ and Membrane Potential in Lymphocytes,” Nature 295 (1982): 68–71. 6799829 10.1038/295068a0

[17] K. Essuman , D. W. Summers , Y. Sasaki , et al., “The SARM1 Toll/Interleukin‐1 Receptor Domain Possesses Intrinsic NAD+ Cleavage Activity That Promotes Pathological Axonal Degeneration,” Neuron 93 (2017): 1334–1343. 28334607 10.1016/j.neuron.2017.02.022 PMC6284238

[18] T. S. Nam , S. H. Choi , S. Y. Rah , et al., “Discovery of a Small‐Molecule Inhibitor for Kidney ADP‐Ribosyl Cyclase: Implication for Intracellular Calcium Signal Mediated by Cyclic ADP‐Ribose,” Experimental & Molecular Medicine 38 (2006): 718–726. 17202848 10.1038/emm.2006.84

[19] K. Venkatachalam , D. B. van Rossum , R. L. Patterson , H. T. Ma , and D. L. Gill , “The Cellular and Molecular Basis of Store‐Operated Calcium Entry,” Nature Cell Biology 4 (2002): E263–E272. 12415286 10.1038/ncb1102-e263

[20] U. H. Kim , “Multiple Enzymatic Activities of CD38 for Ca^2+^ Signaling Messengers,” Messenger 3 (2014): 6–14.

[21] H. Higashida , M. Liang , T. Yoshihara , et al., “An Immunohistochemical, Enzymatic, and Behavioral Study of CD157/BST‐1 as a Neuroregulator,” BMC Neuroscience 18 (2017): 35. 28340569 10.1186/s12868-017-0350-7 PMC5366154

[22] S. Y. Kim , K. H. Park , R. Gul , K. Y. Jang , and U. H. Kim , “Molecular Mechanism of ADP‐Ribosyl Cyclase Activation in Angiotensin II Signaling in Murine Mesangial Cells,” American Journal of Physiology 296 (2009): F291–F297. 18272599 10.1152/ajprenal.00483.2007

