# Supplementary material for: The Na+/K+‐ATPase β1 Subunit is a Kidney ADP‐Ribosyl Cyclase
Source: FASEB J. 2025 Oct 14;39(20):e71139. doi: 10.1096/fj.202502065RR (PMC12519912; doi:10.1096/fj.202502065RR)
Supplement: Supplementary file 1 — Figure S1: Time course of cGDPR production by Atp1b1‐Flag protein. ARC activity was determined by measuring cGDPR using NGD+ as a substrate. Figure S2: ADP‐ribosyl cyclase activities of the purified Atp1b1‐Flag, Sarm1‐Flag and CD38‐Flag proteins. Figure S3: Effect of 4DHAB (200 μM) on ADP‐ribosyl cyclase activity of the purified Atp1b1‐Flag. [file FSB2-39-e71139-s001.docx]

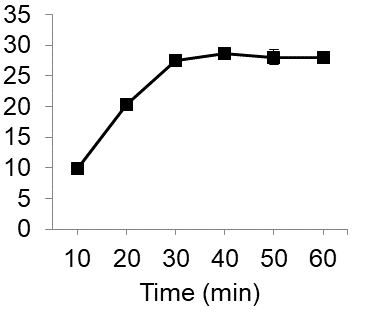


cGDPR Production

(nmol/mg protein)

**FIGURE S1**. Time course of cGDPR production by Atp1b1-Flag protein. ARC activity was determined by measuring cGDPR using NGD^+^ as a substrate.


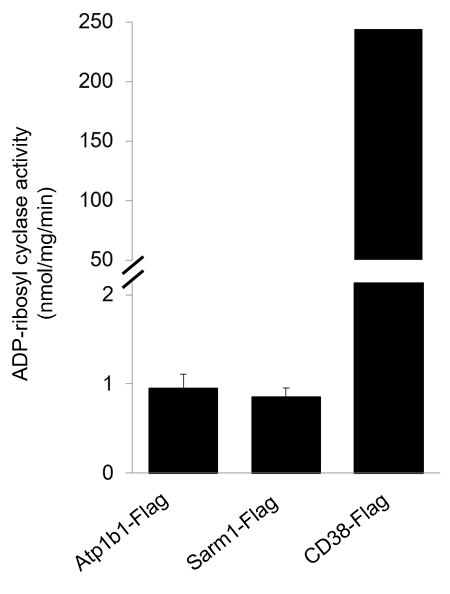


**FIGURE S2**. ADP-ribosyl cyclase activities of the purified Atp1b1-Flag, Sarm1-Flag and CD38-Flag proteins.

**FIGURE S3**. Effect of 4DHAB (200 µM) on ADP-ribosyl cyclase activity of the purified Atp1b1-Flag.
